# Attenuation of partial unilateral ureteral obstruction – induced renal damage with hyperbaric oxygen therapy in a rat model

**DOI:** 10.1590/S1677-5538.IBJU.2016.0565

**Published:** 2017

**Authors:** Eyup Burak Sancak, Yusuf Ziya Tan, Hakan Turkon, Coskun Silan

**Affiliations:** 1 Department of Urology, Canakkale Onsekiz Mart University, Faculty of Medicine, Canakkale, Turkey; 2 Department of Nuclear Medicine, Canakkale Onsekiz Mart University, Faculty of Medicine, Canakkale, Turkey; 3 Department of Biochemistry, Canakkale Onsekiz Mart University, Faculty of Medicine, Canakkale, Turkey; 4 Department of Pharmacology, Canakkale Onsekiz Mart University, Faculty of Medicine, Çanakkale, Turkey

**Keywords:** Hyperbaric Oxygenation, Ureteral Obstruction, Apoptosis

## Abstract

**Objective:**

The objective of the present study was to evaluate the effectiveness of HBO therapy on biochemical parameters, renal morphology and renal scintigraphy in rats undergoing chronic unilateral partial ureteral obstruction (UPUO).

**Material and methods:**

Thirty-five rats were divided into five equal groups: Control group; Sham group; HBO group; UPUO group and UPUO/HBO group. The effects of HBO therapy were examined using biochemical parameters and histopathological changes. After calculating the score for each histopathological change, the total histopathological score was obtained by adding all the scores. In addition, dynamic renal scintigraphy findings were evaluated.

**Results:**

Serum parameters indicating inflammation, serum tumor necrosis factor- alpha, ischemia modified-albumin, IMA/albumin ratio and Pentraxin-3 levels, were observed to be high in the UPUO group and low in the UPUO/HBO treatment group. Similarly, in the treatment group, the reduction in malondialdehyde, total oxidant status and oxidative stress index levels and increase in total antioxidant capacity values were observed to be statistically significant compared to the UPUO group (p<0.001, p=0.007, p<0.001, p=0.001, respectively). The total score and apoptosis index significantly decreased after administration of HBO treatment. Dynamic 99mTc-MAG3 renal scintigraphy also showed convincing evidence regarding the protective nature of HBO against kidney injury. In the UPUO/HBO therapy group, the percentage contribution of each operated kidney increased significantly compared to the UPUO group (41.73% versus 32.72%).

**Conclusion:**

The findings of this study indicate that HBO therapy had a reno-protective effect by reducing inflammation and oxidative stress, and preserving renal function after renal tissue damage due to induction of UPUO.

## INTRODUCTION

Obstructive uropathy is one of the leading causes of end-stage renal disease in children and adults and is associated with increased intraluminal pressure in the ureter and renal tubules that can cause renal parenchymal damage (1). In clinical practice, although unilateral partial ureteral obstruction (UPUO) is considerably more common, the pathophysiologic changes in renal tissue due to UPUO are not understood as well as those in complete ureteral obstruction (2). Known factors in the pathophysiology of the partially obstructed kidney are characterized by renal blood flow impairment, intrapelvic pressure elevation, vasoactive and inflammatory mediators (3). Reactive oxygen species (ROS), which form during ureteral obstruction, are recently recognized as responsible for the development of the pathogenesis of UPUO in experimental research (2, 3). Increased lipid peroxidation and oxidative stress in UPUO have been proposed as possible causes that lead to tubule-interstitial lesions and renal fibrosis. Furthermore, apoptotic cell death has been reported to play an important role in renal damage arising from UPUO (4). Despite advances in supportive precautions and preventive strategies, presently there is no specific medication in clinical use for UPUO-induced renal damage. However, because inflammation, oxidative stress and apoptosis combine in the pathophysiology of UPUO, the optimal therapeutic or preventive approach should target these pathways.

Hyperbaric oxygen (HBO) therapy was first used for the treatment of wounds associated with ischemic tissues (5). HBO therapy involves the intermittent inhalation of 100% oxygen at a pressure higher than 1 standard atmospheric pressure (6). This therapy enhances the dissolved oxygen concentration in arterial blood and restores the diffusion of oxygen into poorly perfused tissues (6, 7). Animal studies have shown that HBO therapy attenuates ischemia reperfusion-induced tissue injury by abating oxidative stress, suppressing inflammation, increasing nitric oxide formation and preventing apoptosis (7-9).

The objective of this study was to evaluate the effectiveness of HBO therapy on biochemical parameters, renal morphology and renal scintigraphy in rats undergoing UPUO.

## MATERIAL AND METHODS

### 

#### Animals and Treatments

Thirty-five male Wistar albino rats, weighing 275±25g, were obtained from the University Experimental Research Center. All rats were placed in separate cages at room temperature during the study under standard laboratory conditions with a 12h dark/12h light cycle. The animals were kept in standard housing conditions with temperature of 25±1°C and 45±5% humidity. The rats were given ad libitum food and water. Sterile conditions were provided during operation.

#### Experimental Protocol

The 35 rats were randomly divided into five equal groups as follows.

Group 1 (Control Group): No procedure administered, taken into the hyperbaric chamber for 14 days but no oxygen administered. On the 14th day under mild ether anesthesia, blood and the left kidney were obtained and rats were sacrificed.

Group 2 (Sham Group): Rats underwent surgical intervention without the final step of partial ureteral ligation. In the 24th hour postoperative the rats were taken to the hyperbaric chamber for one hour but HBO was not administered. The same procedure was repeated on the 14th day postoperative. On the 14th day postoperative renal scintigraphy was performed. Afterwards, blood and tissue samples were taken and rats were sacrificed.

Group 3 (HBO Group): Same procedure as Group 2, however additionally for 14 days HBO therapy was administered.

Group 4 (UPUO Group): Same procedure as Group 2, however UPUO was performed.

Group 5 (UPUO/HBO Group): UPUO was performed. Twenty four hours after operation, the rats were administered HBO. The same procedure was repeated until the 14th day. On the 14th day renal scintigraphy was performed. Afterwards, blood and tissue samples were taken and rats were sacrificed.

#### Technique

Rats anesthetized with 5mg/kg xylazine (Rompun^®^, Bayer, Istanbul, Turkey) and 50mg/ kg ketamine hydrochloride (Ketalar^®^, Eczacibasi, Istanbul, Turkey) with spontaneous respiration at room temperature were placed on a sterile disposable towel over a warming pad. The abdominal skin was prepared with povidone-iodine. After midline incision, the left ureter was located and separated. Psoas muscle was parted 1cm and a tunnel was formed but in Groups 2 and 3 the ureter was not enclosed in the tunnel. In Groups 4 and 5 UPUO was performed according to the technique of Ulm and Miller (10). Psoas muscle was parted 1cm and a tunnel was formed. 1/4 proximal ureter was completely liberated and enclosed in the tunnel. The psoas muscle was closed over the ureter with 6-0 nonabsorbable sutures. The incision was closed with 3-0 silk and rats were returned to cages to recover. The skin was cleaned every day with povidone-iodine to prevent the rats gnawing at the stitches. In Groups 3 and 5, twenty four hours after the operation, the rats were exposed to 100% oxygen for 1 hour at a pressure of 2.5atm in a cylindrical pressure chamber (Barotech Model DB01; BarotechIndustries, Inc., Istanbul, Turkey). In Groups 3 and 5, the final HBO treatment was administered on the postoperative 14th day. One hour after final treatment, the rats in Groups 2, 3, 4 and 5 had renal scintigraphy performed. Immediately afterwards, intracardiac blood samples were obtained and then left nephrectomy was performed immediately. At the end of surgery, all rats were sacrificed by cervical dislocation. Harvested kidneys were divided into two pieces. One piece was put into formalin solution immediately and sent to pathology for histopathological analysis. The others were stored at −800C for biochemical analysis.

#### Laboratory Analysis

Rat tissue and blood samples were obtained at the end of the experiment for each group of animals. The blood samples taken in tubes without anticoagulants were centrifuged at 4000rpm for 10 min for analyses. The resultant serum samples were aliquoted and stored at −80°C until analysis. Albumin, urea, and creatinine levels were determined with a colorimetric method and potassium, chlorine and sodium levels were determined with ion selective electrode methods on the Cobas c501 Autoanalyzer (Roche Diagnostics, Germany).

Serum Pentraxin-3 (PTX3), tumor necrosis factor- alpha (TNF-**α**) and interleukin-6 (IL-6) concentrations were measured with enzyme-linked immunosorbent assay (ELISA) kit (PTX3, Catalog No: CK-E90725, Hangzhou Eastbiopharm Co. Ltd., Hangzhou, China; TNF-**α**, Catalog No: KRC3011; IL-6, Catalog No: KRC0061, Invitrogen Corporation, Camarillo, CA, US) according to the manufacturer's instructions. The intra-assay and inter-assay coefficients of variations were <10 and <12; <6.9 and <9.0; and <5.8% and <8.8 for PTX3, TNF-**α** and IL-6 respectively.

Ischemia-modified albumin (IMA) level was measured by using colorimetric method discovered by Bar-Or et al. (11). The results were reported as absorbance units (ABSUs). Serum IMA/albumin ratio (IMAR) was calculated. Serum IMAR was expressed as Absolute units per gram (ABSU/g) of albumin.

The tissues were prepared at +4°C for biochemical analysis. After washing with phosphate buffer solution (PBS), kidney tissue of the rats was weighed and cut into small pieces, and stored at −80°C in Eppendorf tubes until biochemical analysis. Tissues from all experimental groups were homogenized using Mixer Mill MM 400 (Retsch, Haan, Germany). The protein contents of the tissues were determined according to the method of Lowry et al. (12). Tissue total antioxidant capacity (TAC; Cat. No: RL0017) and total oxidant status (TOS; Cat.No:RL0024) levels were determined with spectrophotometric kits (Rel Assay Diagnostics, Gaziantep, Turkey) as previously described. The TOS/TAC ratio was used to calculate the oxidative stress index (OSI), an indicator of the degree of oxidative stress. Tissue malondialdehyde (MDA) and superoxide dismutase (SOD) levels were measured using commercial available kits according to the manufacturer's directions (OxiSelect MDA and SOD Activity Assay Kits, Cell Biolabs, Cat No: STA-330, STA-340, respectively).

#### Histopathologic evaluation

The removed kidney tissues were immersed in 10% buffered formaldehyde, dehydrated, embedded in paraffin and then cut into 4mm slices. Tissue sections were stained with hematoxylin-eosin (H&E) and periodic acid-schiff (PAS), examined with a Nikon 50i photomicroscope, and photographed with a microscope-connected camera system and APR 50 photos elementary program. Taking into account previous studies, the sections were evaluated and scored for tubular dilatation (TD), interstitial inflammatory cell infiltration (IICI), intertubular hemorrhage and congestion (IHC), interstitial fibrosis (IF), and tubular cast (TC) (13). Tissue damage scores were no visible change [0], minimal or slight change [1], moderate change [2], and severe change [3]. After calculating the score for each of the histopathological changes, the total score was obtained by addition of all the scores from tissue damage markers.

#### Apoptosis evaluation (TUNEL assay)

Apoptotic cells were detected with terminal deoxynucleotidyl-transferase-mediated deoxy-UTP nick end labeling (TUNEL), using an in situ detection kit (ApopTag, Calbiochem, San Diego, CA, USA) according to the manufacturer's instructions. The preparations were investigated at 400 magnification and 6 different areas were screened for each subject. A total of 100 TUNEL-positive or -negative cells were counted in each case, in each different area. For each subject the mean percentage TUNEL-positive cells were taken and the apoptosis index calculated.

#### Dynamic renal scintigraphy

Before imaging, all rats had previously described anesthesia performed. The abdomen was opened along the previous incision line, and ~37MBq/0.2mL 99mTc-MAG3 injection administered intracaval. Immediately after the injection 40 minutes imaging was begun with a Hawkeye dual-head gamma camera (GE Infinia, Buckinghamshire, United Kingdom). In the first minute a total of 60 frames at one second intervals were taken for perfusion images, then a total of 39 frames at one minute intervals were taken for concentration and excretion images. Images were evaluated quantitatively and qualitatively by common consensus of two nuclear medicine experts. The contribution rates for kidneys to total renal function was calculated from regions of interest (ROI) drawn with the aid of a computer. Semi-quantitative evaluation in the Sham/S, UPUO/S and HBO groups assessed the contribution of each kidney to total function. Groups were compared in terms of the percentage contribution of each operated kidney.

### Statistical analysis

Statistical analyses were performed using statistical software SPSS version 15.0 (SPSS Inc, Chicago, IL, USA). Variable distributions were assessed with the Kolmogorov-Smirnov normality test. The results are presented as mean±standard deviation. The significance of the differences between groups was determined using the Student unpaired t-test for normal distribution. Biochemical parameters with non-normal distribution, histopathological findings and scintigraphic results were evaluated with the Kruskal-Wallis test as they were nonparametric. The Mann-Whitney U test was performed to test the significance of pairwise differences using the Bonferroni correction to adjust for multiple comparisons. Values of p<0.05 were considered significant.

## RESULTS

### 

#### Biochemical Findings

Blood biochemical results for the experimental groups are given in Table-1. Between control and sham groups, only IMAR results were significantly different (p=0.045). There was no significant difference between HBO and sham groups in terms of plasma biochemical parameters. When compared with the sham group, the high plasma TNF-α, IMA, IMAR, and PTX3 values in the UPUO group were statistically significant (p=0.045, p=0.028, p=0.011, p=0.045, p=0.035, respectively). In the UPUO/HBO treated group, the plasma TNF-α, IMA, IMAR and PTX3 values were low compared to the UPUO group (p=0.032, p=0.042, p=0.011, p=0.023, respectively).

**Table 1:** Serum biochemical analysis.

	CONTROL	SHAM	HBO	UPUO	UPUO/HBO
Sodium	135.81±2.73	137.26±1.27	136.81±2.62	137.36±1.67	134.52±1.64
Potassium	4.81±0.66	4.65±0.71	4.72±0.79	4.89±0.46	4.92±0.62
Chloride	98.09±2.04	99.08±1.45	99.12±1.72	99.03±2.66	100.69±3.24
Urea	25.36±4.65	29.65±5.76	28.23±12.12	34.53±7.61	30.67±10.97
Creatinine	0.21±0.03	0.26±0.14	0.23±0.15	0.28±0.08	0.26±0.04
Pentraxin-3	1.48±0.34	1.67±0.27	1.66±0.31	2.04±0.30^a^	1.68±0.17^b^
TNF-·α	3.52±0.53	4.30±2.12	3.87±0.91	10.38±6.86^a^	5.67±2.23^b^
IL-6	15.29±3.39	19.09±6.61	17.02±7.29	33.22±24.34	28.52±5.84^a^
IMA	0.64±0.04	0.69±0.05	0.67±0.02	0.82±0.12^a^	0.69±0.06^b^
IMAR	0.17±0.02^a^	0.21±0.03	0.19±0.03	0.32±0.09^a^	0.23±0.03^a^,^b^

Values are mean ± standard deviation. **HBO** = Hyperbaric Oxygen Group; **UPUO** = unilateral partial ureteral obstruction group; **UPUO/HBO** = UPUO and HBO treated group; **TNF-α** = Tumor necrosis factor- alpha; **IL-6** = interleukin-6; **IMA** = Ischemia modified albumin; **IMAR** = IMA/albumin ratio.

a p< 0.05 compared with the SHAM group;

b p< 0.05 compared with the UPUO group

Tissue biochemical results for the groups are given in Table-2. According to tissue biochemistry results, there was no significant difference between the control and HBO groups with the sham group in terms of the parameters. Compared with the sham group, the UPUO group had high MDA, TOS and OSI values and low TAC value that were statistically significant (p=0.010, p=0.004, p<0.001, p=0.001, respectively). In the treatment group, the reduction in MDA, TOS and OSI values and increase in TAC values were observed to be statistically significant compared to the UPUO group (p<0.001, p=0.007, p<0.001, p=0.001, respectively).

**Table 2:** Tissue biochemical analysis.

	CONTROL	SHAM	HBO	UPUO	UPUO/HBO
SOD	3.78±1.74	3.35±1.70	3.55±1.59	1.89±0.97	2.63±0.93
MDA	1.09±0.38	1.31±0.45	0.96±0.32	2.04±0.46^a^	1.06±0.30^b^
TAC	0.37±0.20	0.32±0.06	0.35±0.04	0.19±0.05^a^	0.31±0.03^b^
TOS	0.86±0.34	0.98±0.48	0.94±0.41	2.07±0.67^a^	1.01±0.54^b^
OSI	2.80±0.83	3.08±1.65	2.66±1.08	8.64±2.18^a^	3.79±1.95^b^

Values are mean ± standard deviation. **HBO** = Hyperbaric Oxygen Group; **UPUO** = Unilateral partial ureteral obstruction group; **UPUO/HBO** = UPUO and HBO treated group; **TNF-α** = Tumor necrosis factor- alpha; **IL-6** = interleukin-6; **IMA** = Ischemia modified albumin; **IMAR** = IMA/albumin ratio; **SOD** = Superoxide dismutase (U/g wet tissue.); **MDA** = Malondialdehyde (μΜ/g wet tissue); **TAC** = Total antioxidant capacity (mmol Trolox Equiv./g wet tissue); **TOS** = Total oxidant status (·μmol H_2_O_2_ Equiv./g wet tissue); **OSI** = Oxidative stress index (arbitrary unit).

a p< 0.05 compared with the SHAM group;

b p< 0.05 compared with the UPUO group

#### Histopathologic Findings

**Figure 1:**
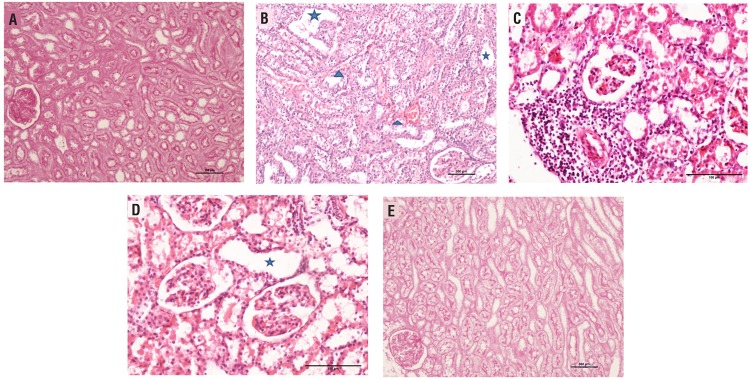
a) Normal histological structure in the sham group, regular edges observed [Periodic acid-schiff (PASx100)]. b) Tubular dilatation (TD) (★), intertubular hemorrhage and congestion (IHC) (▲) and disrupted glomerular structures observed in the unilateral partial ureteral obstruction (UPUO) [Hematoxylin-eosin (H&Ex200)]. c) Clear infiltration by interstitial inflammatory cells in the UPUO group (H&Ex200). d) TD (★) and tubular cast (TC) observed in the UPUO group (H&Ex200) e: Close to normal histological structure, regular brush-like edges in the UPUO/HBO therapy group. (PASx100).

Histopathological analysis and apoptosis index results for the groups are given in Table-3. There was no significant difference in the parameters between the control and HBO groups and the sham group. The TD, IICI, IHC, IF and TC scores in the UPUO group were observed to be higher than the sham group (p=0.001, p=0.001, p=0.001, p=0.001, p=0.011, respectively). In the UPUO/HBO treatment group, the mean TD, IICI, IHC and IF pathological evaluation scores were identified to be low compared to the UPUO group (p=0.002, p=0.002, p=0.026, p=0.002). Obtained by summing all the scores from morphological renal damage markers, the total score was increased in the UPUO group when compared with the Sham group (11.14±2.55, 1.43±1.90, p=0.001). But, the total score significantly decreased after administration of HBO (4.14±1.77, p=0.001 compared with the UPUO group) (Figure-1).

**Table 3:** Histopathological Analysis and Apoptosis Index Results.

GROUPS	TD	IICI	IHC	IF	TC	TOTAL NUMBER	APOPTOSIS
CONTROL	0.29±0.49	0.14±0.38	0.14±0.38	0.14±0.38	0.14±0.38	0.86±0.69	2.31±0.38
SHAM	0.43±0.54	0.29±0.49	0.29±0.49	0.14±0.38	0.29±0.49	1.43±1.90	2.87±0.98
HBO	0.14±0.38	0.29±0.49	0.43±0.54	0.14±0.38	0.14±0.38	1.14±1.07	2.45±0.53
UPUO	2.43±0.54^a^	2.71±0.49^a^	2.29±0.76^a^	1.43±0.98^a^	1.29±0.76^a^	11.14±2.55^a^	27.85±7.78^a^
UPUO/HBO	1.00±0.58^b^	1.14±0.69^a^,^b^	1.14±0.69^a^,^b^	0.33±0.54^b^	0.43±0.54	4.14±1.77^b^	9.00±2.94^a^,^b^

Values are mean ± standard deviation. **TD** = Tubular dilatation; **IICI** = Interstitial inflammatory cells infiltration; **IHC** = Intertubular hemorrhage and congestion; **IF** = Interstitial fibrosis; **TC** = tubular cast; **HBO** = Hyperbaric Oxygen Group; **UPUO** = unilateral partial ureteral obstruction group; **UPUO/HBO** = UPUO and HBO treated group.

a p< 0.05 compared with Sham group;

b p< 0.05 compared with UPUO group.

**Figure 2:**
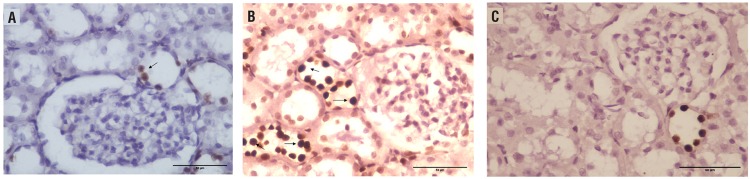
Apoptotic cells were detected with the TUNEL method (400X). a) Low numbers of TUNEL (+) apoptotic cells in the sham group. b) Chronic obstructive tissue in UPUO group has dense apoptosis. c) Apoptotic cells are diminished in the UPUO/HBO therapy group.

Apoptosis Index observed using immunohistochemical staining was 2.87±0.98 in the Sham group, 27.85 in the UPUO group (p=0.001 compared with the Sham group) and 9.0 in the UPUO/HBO group (p=0.001 compared with the UPUO group) (Figure-2).

#### Renal Scintigraphy Findings

**Figure 3:**
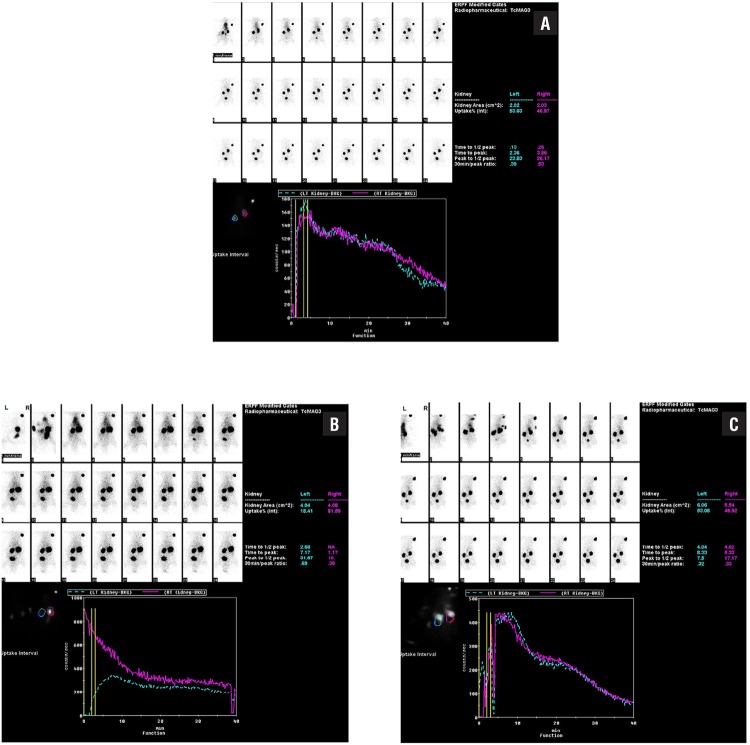
Dynamic renal scintigraphy with 99mTc-MAG3. a) Functions of both kidneys appear normal in the sham group. b) Chronic partial obstructed left kidney in UPUO group has low percentage contribution. c) Close to normal percentage of uptake observed in the UPUO/HBO therapy group (For orientation during imaging, an external marker was placed on the right side of the rat's head).

Dynamic renal scintigraphy with 99mTc - MAG3 results for the groups are given in Table-4. The average of percentage contribution by the intervened kidney was 48.66±1.57 in the Sham group, 32.72±4.17 in the UPUO group (p<0.001 compared with the Sham group) and 41.73±3.00 in the UPUO/HBO group (p=0.001 compared with the UPUO group) (Figure-3). There was no significant difference between the Sham and HBO groups for dynamic renal scintigraphy results.

**Table 4:** Dynamic renal scintigraphy with 99mTc-MAG3 showing residual functional capacity of injured kidney.

	SHAM	HBO	UPUO	UPUO/HBO
Mean %	48.66±1.57	49.80±2.34	32.72±4.17^a^	41.73±3.00^a^,^b^

Values are mean ± standard deviation. **HBO** = Hyperbaric Oxygen Group; **UPUO** = unilateral partial ureteral obstruction group; **UPUO/HBO** = UPUO and HBO treated group

a p< 0.05 compared with Sham group;

b p< 0.05 compared with UPUO group.

## DISCUSSION

This is the first study that demonstrates the efficiency of HBO therapy on biochemical parameters, renal morphology and renal scintigraphy in rats undergoing UPUO. Our study demonstrated that HBO therapy had a reno-protective effect by reducing inflammation and oxidative stress, and preserving renal function in renal tissue injury after the induction of UPUO.

Obstructive uropathy is a common problem in daily clinical urology practice and can occur anywhere along the urinary tract (14). Upper urinary tract obstruction is most often secondary to calculi, strictures, tumors and ureteropelvic junction or ureterovesical junction obstruction and is usually a reversible and partial unilateral condition (4). Although ureteral obstructions may cause kidney parenchymal damage due to several mechanisms, the exact pathophysiological mechanism of the changes in UPUO has not been fully understood (3, 14). Intrarenal collecting system pressure elevation, renal blood flow impairment, vasoactive and inflammatory mediators are some of the known factors in pathophysiology of renal damage arising from UPUO (3, 4). As renal blood flow increases in the first 12 hour period after obstruction, hypoperfusion develops in advancing periods and is called secondary vascular injury. In current publications it is reported that ROS may lead to severe injury to the cell membrane by lipid peroxidation reactions and may have an important role in tubulointerstitial inflammation associated with obstructive nephropathy (3, 4, 14). Additionally, apoptosis has been reported to play an important role in pathophysiology of obstructive renal parenchymal injury (4).

Hyperbaric oxygen therapy is defined as respiration of 100% oxygen by patients at intervals in a pressurized treatment room with pressure higher than sea level (15). Currently HBO is used as alternative treatment for ischemic injury to renal, brain, pulmonary, liver, cardiac, skeletal muscle, testes and intestinal tissue (7). It has been shown to have anti-inflammatory and anti-oxidant effects on ischemic tissue, reducing neutrophil adhesion, free radical production and apoptosis and increasing nitric oxide production (16).

Some studies have shown that HBO therapy improved the glomerular filtration rate (GFR) in kidneys injured after renal ischemic reperfusion (17). Additionally, HBO therapy increases both vasoconstriction and tissue oxygenation, and is the only treatment modality causing hyperoxic vasoconstriction (18). With all these properties, HBO therapy is expected to provide positive contribution to both primary and secondary injury periods in renal tissue damage as a result of chronic obstruction.

The chain of inflammatory events caused by UPUO includes infiltration of the kidney by inflammatory cells involving monocytes, activation, and possible transformation of intrinsic renal cells, and interactions between infiltrating and resident cells (1). A study by Solmazgül et al. stated that HBO reduced neutrophil infiltration in rats with renal ischemia reperfusion (19). Another study reported that HBO had an anti-inflammatory effect with decreased neutrophil activation and downregulation of adhesion molecule expression on endothelial cells, precluding adherence between neutrophils and endothelial cells (1).

The long PTX3 protein is a member of a superfamily of conserved proteins, and is expressed in vascular endothelial cells and macrophages. Thus, its levels may more directly reflect the inflammatory condition of the vasculature (20). Recently, several clinical investigations have demonstrated that higher plasma PTX3 levels are associated with cardiovascular diseases and with lower GFR and independently predict incidence of chronic kidney diseases (20, 21). In our study, in accordance with the literature, plasma PTX3 levels were significantly higher in the chronic UPUO group than in the Sham group. In the UPUO/HBO treatment group, however, these parameters decreased compared to the UPUO group. Other parameters indicating inflammation, similar to TNF-**α**, IMA and IMAR values, were observed to be high in the chronic UPUO group and low in the UPUO/ HBO treatment group.

There has been concern about the use of HBO, based on the hypothesis that providing extra oxygen would increase free radical production and tissue damage. However, HBO has been shown to decrease lipid peroxidation in a number of studies (8, 18, 22). A study by Ilhan et al. determined that HBO therapy attenuated MDA levels by increasing SOD and GPx activities in rats with renal ischemia reperfusion (6). In recent years, diverse oxidant species can be measured totally and lipid peroxidation activities have been analyzed by evaluating TOS (23). The protecting enzymes from oxidative stress of SOD, catalase (CAT), glutathione reductase (GR) and glutathione peroxidase (GPx) resist the destructive actions of ROS, and these enzymes engender the TAC. The advantage of TAC measurement is that it can measure the antioxidant capacity of all antioxidants in a biological sample. Even more importantly, the ratio of TOS to TAC is accepted as the OSI, which is a more valuable indicator to reflect oxidative status than TAC or TOS level alone (13, 23). In this study, we found that tissue MDA, TOS and OSI levels were significantly higher in the UPUO group than in the Sham group. After treatment with HBO, MDA, TOS and OSI levels were markedly decreased. Although TAC and SOD levels were lower in the UPUO group and higher in the UPUO/HBO group, there were no significant changes detected in SOD values between the groups. The reason for these results can be attributed as being due to the 14-day chronic process possibly reducing the endogenous SOD activity. According to blood and tissue biochemical findings, HBO was observed to have a renoprotective effect reducing inflammation and oxidative stress in chronic UPUO.

Histopathologic findings also showed the protective nature of HBO against kidney injury induced during chronic UPUO. Migita et al. demonstrated that HBO suppressed apoptosis, and promoted tubular cell regeneration after renal ischemia/reperfusion injury (IRI) in rats (9). Gurer et al. showed that HBO therapy attenuated renal IRI in rats by decreasing total histopathology scores (8). In accordance with the literature, in our study the apoptosis index decreased significantly in the UPUO/HBO group compared with the UPUO group. The total histopathologic score was higher in the UPUO group compared with the Sham group. It was observed that the total score significantly decreased in the group administered HBO therapy. Briefly, amelioration was detected in the UPUO/ HBO treatment group in the form of a remarkable decline in histopathological changes caused by chronic UPUO. In addition, the components of nephrons, the glomeruli and tubules were observed to be apparently healthy.

In the study by Rubinstein et al. they demonstrated that HBO treatment has potential in the treatment of GFR by improving the antioxidant/oxidant balance in the ischemic kidney (17). Accordingly, GFR was reduced by 94% in the untreated group compared with the untouched normal kidney. In contrast, in the HBO therapy group, GFR of the ischemic kidney was reduced only by 68%. In the literature we did not encounter any other study evaluating the effect of HBO with renal scintigraphy. In our study on dynamic renal scintigraphy with 99mTc-MAG3, the percentage contribution of each intervened kidney was calculated to be 48.66 in the sham group, 32.72 in the UPUO group and 41.73 in the UPUO/HBO group.

Factors limiting our study include the fact that though HBO therapy suppresses inflammation increasing NO formation, in our study NO levels were not examined. Again, enzymes with very short half lives like SOD and MDA were no evaluated during the study period.

## CONCLUSIONS

The findings of this study indicate that HBO administration improves impairment of renal functions in rats experiencing UPUO. HBO therapy diminished the inflammation parameters in serum and oxidative stress parameters in tissue, and ameliorated histopathological alterations and apoptosis. Dynamic renal scintigraphy also showed convincing evidence regarding the protective nature of HBO against kidney injury. More studies should be conducted to provide a better understanding of the potential benefits and fully assess the effect of HBO on chronic obstructive kidney damage.
